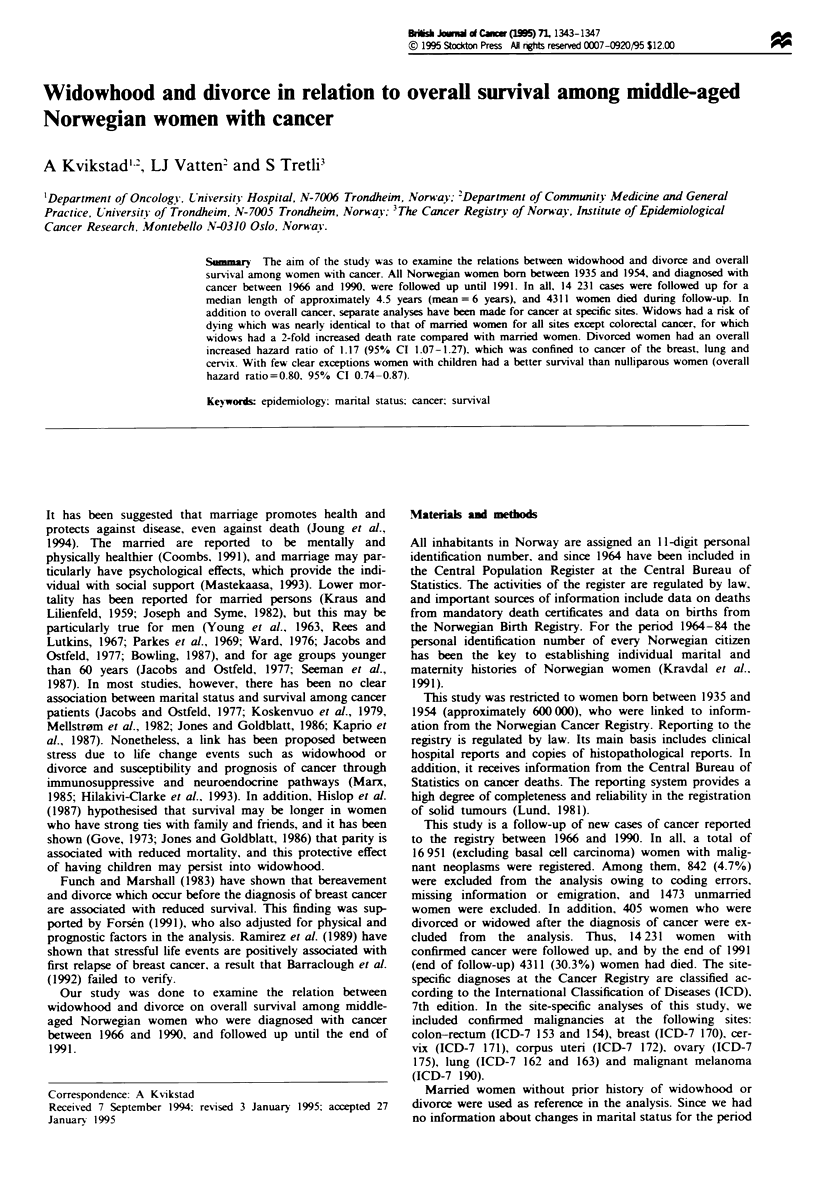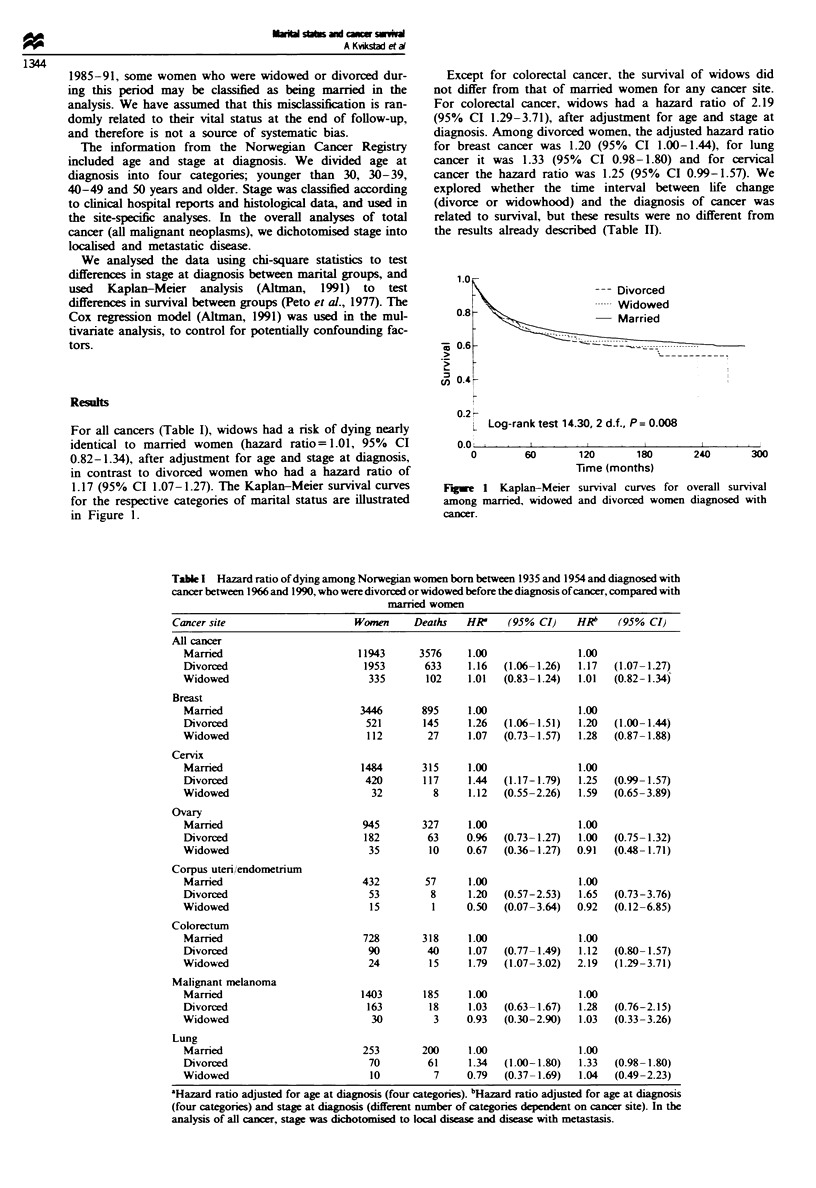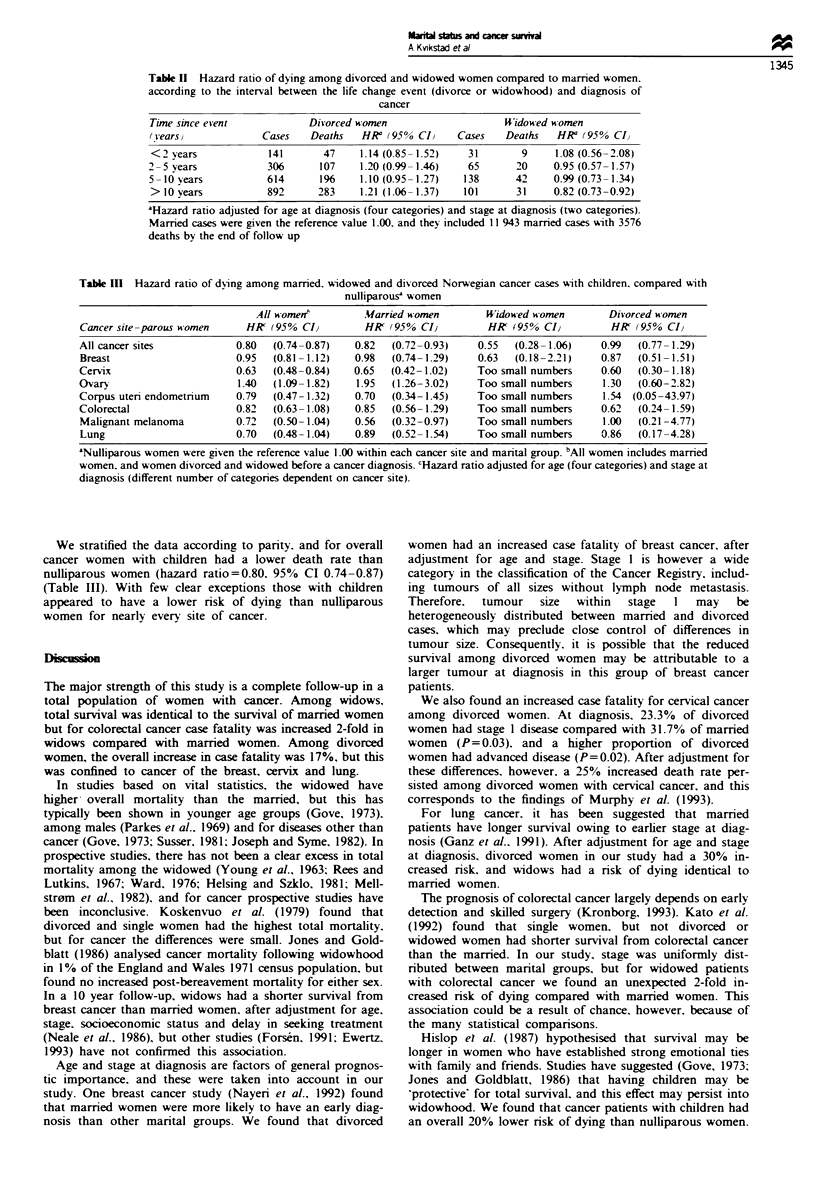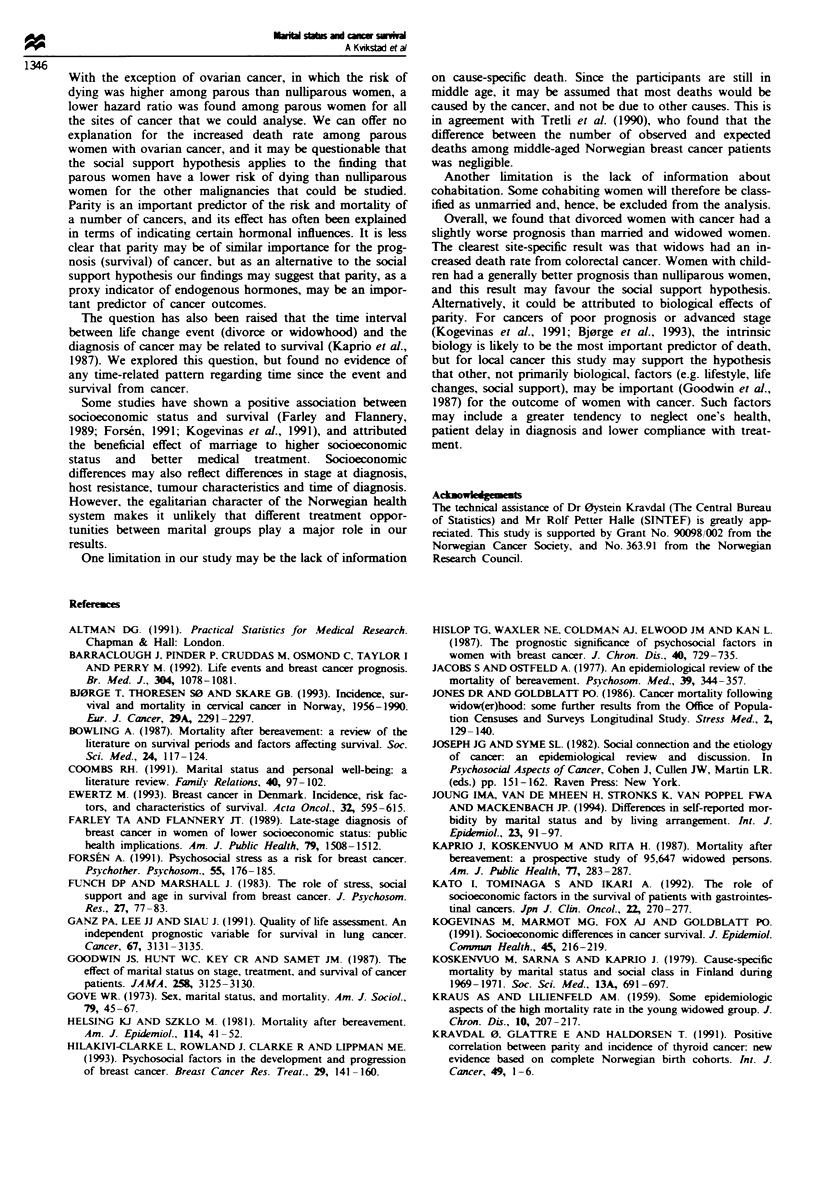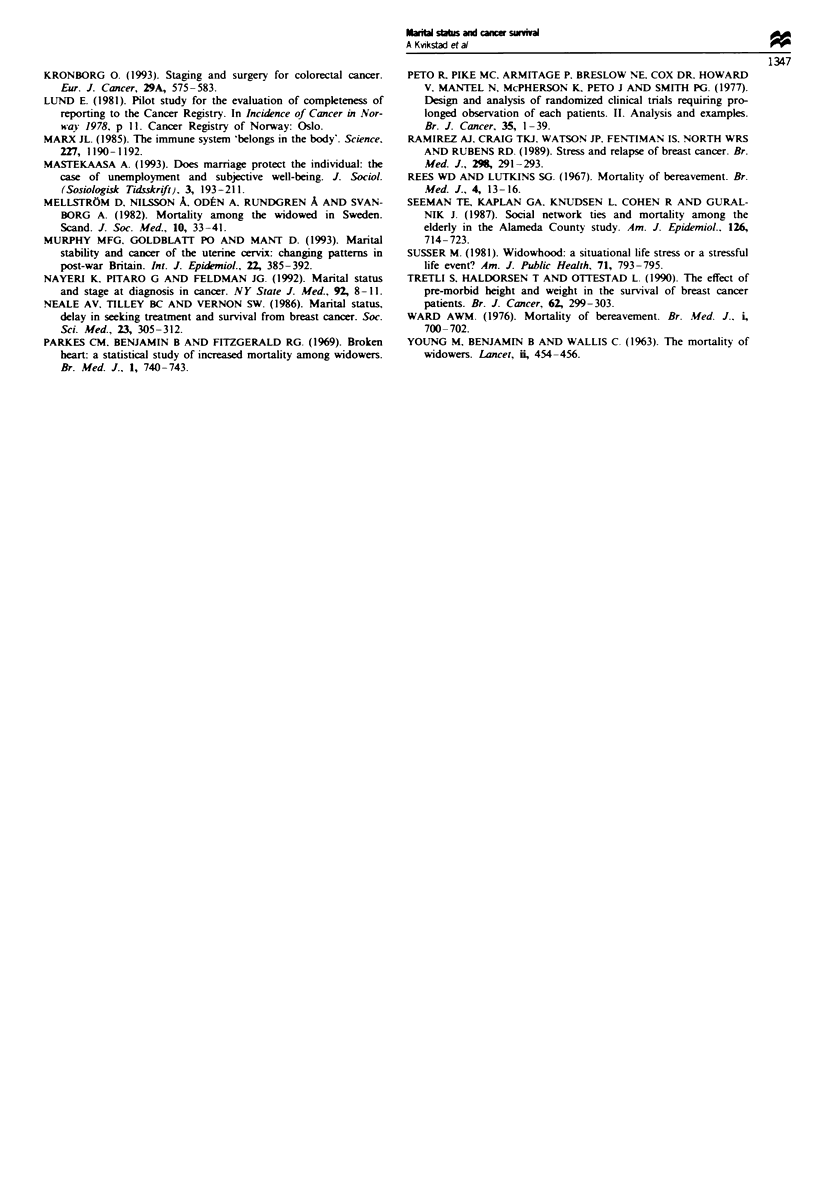# Widowhood and divorce in relation to overall survival among middle-aged Norwegian women with cancer.

**DOI:** 10.1038/bjc.1995.261

**Published:** 1995-06

**Authors:** A. Kvikstad, L. J. Vatten, S. Tretli

**Affiliations:** Department of Oncology, University Hospital, Trondheim, Norway.

## Abstract

The aim of the study was to examine the relations between widowhood and divorce and overall survival among women with cancer. All Norwegian women born between 1935 and 1954, and diagnosed with cancer between 1966 and 1990, were followed up until 1991. In all, 14,231 cases were followed up for a median length of approximately 4.5 years (mean = 6 years), and 4311 women died during follow-up. In addition to overall cancer, separate analyses have been made for cancer at specific sites. Widows had a risk of dying which was nearly identical to that of married women for all sites except colorectal cancer, for which widows had a 2-fold increased death rate compared with married women. Divorced women had an overall increased hazard ratio of 1.17 (95% CI 1.07-1.27), which was confined to cancer of the breast, lung and cervix. With few clear exceptions women with children had a better survival than nulliparous women (overall hazard ratio = 0.80, 95% CI 0.74-0.87).


British Journal of Cancer (1995) 71, 1343-1347

© 1995 Stockton Press All rights reserved 0007-0920/95 $12.00

### Widowhood and divorce in relation to overall survival among middle-aged Norwegian women with cancer

A Kvikstad[1,2], LJ Vatten[2] and S Tretli[3]

[1]*Department of Oncology, University Hospital, N-7006 Trondheim, Norway;* [2]*Department of Community Medicine and General Practice, University of Trondheim, N-7005 Trondheim, Norway;* [3]*The Cancer Registry of Norway, Institute of Epidemiological Cancer Research, Montebello N-0310 Oslo, Norway.*

**Summary** The aim of the study was to examine the relations between widowhood and divorce and overall survival among women with cancer. All Norwegian women born between 1935 and 1954, and diagnosed with cancer between 1966 and 1990, were followed up until 1991. In all, 14 231 cases were followed up for a median length of approximately 4.5 years (mean = 6 years), and 4311 women died during follow-up. In addition to overall cancer, separate analyses have been made for cancer at specific sites. Widows had a risk of dying which was nearly identical to that of married women for all sites except colorectal cancer, for which widows had a 2-fold increased death rate compared with married women. Divorced women had an overall increased hazard ratio of 1.17 (95% CI 1.07-1.27), which was confined to cancer of the breast, lung and cervix. With few clear exceptions women with children had a better survival than nulliparous women (overall hazard ratio = 0.80, 95% CI 0.74-0.87).

**Keywords:** epidemiology; marital status; cancer; survival

It has been suggested that marriage promotes health and protects against disease, even against death (Joung *et al.*, 1994). The married are reported to be mentally and physically healthier (Coombs, 1991), and marriage may particularly have psychological effects, which provide the individual with social support (Mastekaasa, 1993). Lower mortality has been reported for married persons (Kraus and Lilienfeld, 1959; Joseph and Syme, 1982), but this may be particularly true for men (Young *et al.*, 1963, Rees and Lutkins, 1967; Parkes *et al.*, 1969; Ward, 1976; Jacobs and Ostfeld, 1977; Bowling, 1987), and for age groups younger than 60 years (Jacobs and Ostfeld, 1977; Seeman *et al.*, 1987). In most studies, however, there has been no clear association between marital status and survival among cancer patients (Jacobs and Ostfeld, 1977; Koskenvuo *et al.*, 1979, Mellstrøm *et al.*, 1982; Jones and Goldblatt, 1986; Kaprio *et al.*, 1987). Nonetheless, a link has been proposed between stress due to life change events such as widowhood or divorce and susceptibility and prognosis of cancer through immunosuppressive and neuroendocrine pathways (Marx, 1985; Hilakivi-Clarke *et al.*, 1993). In addition, Hislop *et al.* (1987) hypothesised that survival may be longer in women who have strong ties with family and friends, and it has been shown (Gove, 1973; Jones and Goldblatt, 1986) that parity is associated with reduced mortality, and this protective effect of having children may persist into widowhood.

Funch and Marshall (1983) have shown that bereavement and divorce which occur before the diagnosis of breast cancer are associated with reduced survival. This finding was supported by Forsén (1991), who also adjusted for physical and prognostic factors in the analysis. Ramirez *et al.* (1989) have shown that stressful life events are positively associated with first relapse of breast cancer, a result that Barraclough *et al.* (1992) failed to verify.

Our study was done to examine the relation between widowhood and divorce on overall survival among middle-aged Norwegian women who were diagnosed with cancer between 1966 and 1990, and followed up until the end of 1991.

Correspondence: A Kvikstad
Received 7 September 1994; revised 3 January 1995; accepted 27 January 1995

### Materials and methods

All inhabitants in Norway are assigned an 11-digit personal identification number, and since 1964 have been included in the Central Population Register at the Central Bureau of Statistics. The activities of the register are regulated by law, and important sources of information include data on deaths from mandatory death certificates and data on births from the Norwegian Birth Registry. For the period 1964-84 the personal identification number of every Norwegian citizen has been the key to establishing individual marital and maternity histories of Norwegian women (Kravdal *et al.*, 1991).

This study was restricted to women born between 1935 and 1954 (approximately 600 000), who were linked to information from the Norwegian Cancer Registry. Reporting to the registry is regulated by law. Its main basis includes clinical hospital reports and copies of histopathological reports. In addition, it receives information from the Central Bureau of Statistics on cancer deaths. The reporting system provides a high degree of completeness and reliability in the registration of solid tumours (Lund, 1981).

This study is a follow-up of new cases of cancer reported to the registry between 1966 and 1990. In all, a total of 16 951 (excluding basal cell carcinoma) women with malignant neoplasms were registered. Among them, 842 (4.7%) were excluded from the analysis owing to coding errors, missing information or emigration, and 1473 unmarried women were excluded. In addition, 405 women who were divorced or widowed after the diagnosis of cancer were excluded from the analysis. Thus, 14 231 women with confirmed cancer were followed up, and by the end of 1991 (end of follow-up) 4311 (30.3%) women had died. The site-specific diagnoses at the Cancer Registry are classified according to the International Classification of Diseases (ICD), 7th edition. In the site-specific analyses of this study, we included confirmed malignancies at the following sites: colon-rectum (ICD-7 153 and 154), breast (ICD-7 170), cervix (ICD-7 171), corpus uteri (ICD-7 172), ovary (ICD-7 175), lung (ICD-7 162 and 163) and malignant melanoma (ICD-7 190).

Married women without prior history of widowhood or divorce were used as reference in the analysis. Since we had no information about changes in marital status for the period

1985–91, some women who were widowed or divorced during this period may be classified as being married in the analysis. We have assumed that this misclassification is randomly related to their vital status at the end of follow-up, and therefore is not a source of systematic bias.

The information from the Norwegian Cancer Registry included age and stage at diagnosis. We divided age at diagnosis into four categories; younger than 30, 30–39, 40–49 and 50 years and older. Stage was classified according to clinical hospital reports and histological data, and used in the site-specific analyses. In the overall analyses of total cancer (all malignant neoplasms), we dichotomised stage into localised and metastatic disease.

We analysed the data using chi-square statistics to test differences in stage at diagnosis between marital groups, and used Kaplan–Meier analysis (Altman, 1991) to test differences in survival between groups (Peto *et al.*, 1977). The Cox regression model (Altman, 1991) was used in the multivariate analysis, to control for potentially confounding factors.

### Results

For all cancers (Table I), widows had a risk of dying nearly identical to married women (hazard ratio = 1.01, 95% CI 0.82–1.34), after adjustment for age and stage at diagnosis, in contrast to divorced women who had a hazard ratio of 1.17 (95% CI 1.07–1.27). The Kaplan–Meier survival curves for the respective categories of marital status are illustrated in Figure 1.

Except for colorectal cancer, the survival of widows did not differ from that of married women for any cancer site. For colorectal cancer, widows had a hazard ratio of 2.19 (95% CI 1.29–3.71), after adjustment for age and stage at diagnosis. Among divorced women, the adjusted hazard ratio for breast cancer was 1.20 (95% CI 1.00–1.44), for lung cancer it was 1.33 (95% CI 0.98–1.80) and for cervical cancer the hazard ratio was 1.25 (95% CI 0.99–1.57). We explored whether the time interval between life change (divorce or widowhood) and the diagnosis of cancer was related to survival, but these results were no different from the results already described (Table II).

**Figure 1** Kaplan–Meier survival curves for overall survival among married, widowed and divorced women diagnosed with cancer.

**Table I** Hazard ratio of dying among Norwegian women born between 1935 and 1954 and diagnosed with cancer between 1966 and 1990, who were divorced or widowed before the diagnosis of cancer, compared with married women

| *Cancer site* | *Women* | *Deaths* | *HR*[a] | *(95% CI)* | *HR*[b] | *(95% CI)* |
|---|---|---|---|---|---|---|
| All cancer | | | | | | |
| Married | 11943 | 3576 | 1.00 | | 1.00 | |
| Divorced | 1953 | 633 | 1.16 | (1.06–1.26) | 1.17 | (1.07–1.27) |
| Widowed | 335 | 102 | 1.01 | (0.83–1.24) | 1.01 | (0.82–1.34) |
| Breast | | | | | | |
| Married | 3446 | 895 | 1.00 | | 1.00 | |
| Divorced | 521 | 145 | 1.26 | (1.06–1.51) | 1.20 | (1.00–1.44) |
| Widowed | 112 | 27 | 1.07 | (0.73–1.57) | 1.28 | (0.87–1.88) |
| Cervix | | | | | | |
| Married | 1484 | 315 | 1.00 | | 1.00 | |
| Divorced | 420 | 117 | 1.44 | (1.17–1.79) | 1.25 | (0.99–1.57) |
| Widowed | 32 | 8 | 1.12 | (0.55–2.26) | 1.59 | (0.65–3.89) |
| Ovary | | | | | | |
| Married | 945 | 327 | 1.00 | | 1.00 | |
| Divorced | 182 | 63 | 0.96 | (0.73–1.27) | 1.00 | (0.75–1.32) |
| Widowed | 35 | 10 | 0.67 | (0.36–1.27) | 0.91 | (0.48–1.71) |
| Corpus uteri/endometrium | | | | | | |
| Married | 432 | 57 | 1.00 | | 1.00 | |
| Divorced | 53 | 8 | 1.20 | (0.57–2.53) | 1.65 | (0.73–3.76) |
| Widowed | 15 | 1 | 0.50 | (0.07–3.64) | 0.92 | (0.12–6.85) |
| Colorectum | | | | | | |
| Married | 728 | 318 | 1.00 | | 1.00 | |
| Divorced | 90 | 40 | 1.07 | (0.77–1.49) | 1.12 | (0.80–1.57) |
| Widowed | 24 | 15 | 1.79 | (1.07–3.02) | 2.19 | (1.29–3.71) |
| Malignant melanoma | | | | | | |
| Married | 1403 | 185 | 1.00 | | 1.00 | |
| Divorced | 163 | 18 | 1.03 | (0.63–1.67) | 1.28 | (0.76–2.15) |
| Widowed | 30 | 3 | 0.93 | (0.30–2.90) | 1.03 | (0.33–3.26) |
| Lung | | | | | | |
| Married | 253 | 200 | 1.00 | | 1.00 | |
| Divorced | 70 | 61 | 1.34 | (1.00–1.80) | 1.33 | (0.98–1.80) |
| Widowed | 10 | 7 | 0.79 | (0.37–1.69) | 1.04 | (0.49–2.23) |

[a]Hazard ratio adjusted for age at diagnosis (four categories). [b]Hazard ratio adjusted for age at diagnosis (four categories) and stage at diagnosis (different number of categories dependent on cancer site). In the analysis of all cancer, stage was dichotomised to local disease and disease with metastasis.

**Table II** Hazard ratio of dying among divorced and widowed women compared to married women, according to the interval between the life change event (divorce or widowhood) and diagnosis of cancer

| *Time since event (years)* | *Divorced women* Cases | Deaths | HR[a] (95% CI) | *Widowed women* Cases | Deaths | HR[a] (95% CI) |
|---|---|---|---|---|---|---|
| <2 years | 141 | 47 | 1.14 (0.85-1.52) | 31 | 9 | 1.08 (0.56-2.08) |
| 2-5 years | 306 | 107 | 1.20 (0.99-1.46) | 65 | 20 | 0.95 (0.57-1.57) |
| 5-10 years | 614 | 196 | 1.10 (0.95-1.27) | 138 | 42 | 0.99 (0.73-1.34) |
| >10 years | 892 | 283 | 1.21 (1.06-1.37) | 101 | 31 | 0.82 (0.73-0.92) |

[a]Hazard ratio adjusted for age at diagnosis (four categories) and stage at diagnosis (two categories). Married cases were given the reference value 1.00, and they included 11 943 married cases with 3576 deaths by the end of follow up

**Table III** Hazard ratio of dying among married, widowed and divorced Norwegian cancer cases with children, compared with nulliparous[a] women

| *Cancer site-parous women* | *All women[b]* HR[c] | (95% CI) | *Married women* HR[c] | (95% CI) | *Widowed women* HR[c] | (95% CI) | *Divorced women* HR[c] | (95% CI) |
|---|---|---|---|---|---|---|---|---|
| All cancer sites | 0.80 | (0.74-0.87) | 0.82 | (0.72-0.93) | 0.55 | (0.28-1.06) | 0.99 | (0.77-1.29) |
| Breast | 0.95 | (0.81-1.12) | 0.98 | (0.74-1.29) | 0.63 | (0.18-2.21) | 0.87 | (0.51-1.51) |
| Cervix | 0.63 | (0.48-0.84) | 0.65 | (0.42-1.02) | Too small numbers | | 0.60 | (0.30-1.18) |
| Ovary | 1.40 | (1.09-1.82) | 1.95 | (1.26-3.02) | Too small numbers | | 1.30 | (0.60-2.82) |
| Corpus uteri endometrium | 0.79 | (0.47-1.32) | 0.70 | (0.34-1.45) | Too small numbers | | 1.54 | (0.05-43.97) |
| Colorectal | 0.82 | (0.63-1.08) | 0.85 | (0.56-1.29) | Too small numbers | | 0.62 | (0.24-1.59) |
| Malignant melanoma | 0.72 | (0.50-1.04) | 0.56 | (0.32-0.97) | Too small numbers | | 1.00 | (0.21-4.77) |
| Lung | 0.70 | (0.48-1.04) | 0.89 | (0.52-1.54) | Too small numbers | | 0.86 | (0.17-4.28) |

[a]Nulliparous women were given the reference value 1.00 within each cancer site and marital group. [b]All women includes married women, and women divorced and widowed before a cancer diagnosis. [c]Hazard ratio adjusted for age (four categories) and stage at diagnosis (different number of categories dependent on cancer site).

We stratified the data according to parity, and for overall cancer women with children had a lower death rate than nulliparous women (hazard ratio=0.80, 95% CI 0.74-0.87) (Table III). With few clear exceptions those with children appeared to have a lower risk of dying than nulliparous women for nearly every site of cancer.

### Discussion

The major strength of this study is a complete follow-up in a total population of women with cancer. Among widows, total survival was identical to the survival of married women but for colorectal cancer case fatality was increased 2-fold in widows compared with married women. Among divorced women, the overall increase in case fatality was 17%, but this was confined to cancer of the breast, cervix and lung.

In studies based on vital statistics, the widowed have higher overall mortality than the married, but this has typically been shown in younger age groups (Gove, 1973), among males (Parkes *et al.*, 1969) and for diseases other than cancer (Gove, 1973; Susser, 1981; Joseph and Syme, 1982). In prospective studies, there has not been a clear excess in total mortality among the widowed (Young *et al.*, 1963; Rees and Lutkins, 1967; Ward, 1976; Helsing and Szklo, 1981; Mellstrøm *et al.*, 1982), and for cancer prospective studies have been inconclusive. Koskenvuo *et al.* (1979) found that divorced and single women had the highest total mortality, but for cancer the differences were small. Jones and Goldblatt (1986) analysed cancer mortality following widowhood in 1% of the England and Wales 1971 census population, but found no increased post-bereavement mortality for either sex. In a 10 year follow-up, widows had a shorter survival from breast cancer than married women, after adjustment for age, stage, socioeconomic status and delay in seeking treatment (Neale *et al.*, 1986), but other studies (Forsén, 1991; Ewertz, 1993) have not confirmed this association.

Age and stage at diagnosis are factors of general prognostic importance, and these were taken into account in our study. One breast cancer study (Nayeri *et al.*, 1992) found that married women were more likely to have an early diagnosis than other marital groups. We found that divorced women had an increased case fatality of breast cancer, after adjustment for age and stage. Stage 1 is however a wide category in the classification of the Cancer Registry, including tumours of all sizes without lymph node metastasis. Therefore, tumour size within stage 1 may be heterogeneously distributed between married and divorced cases, which may preclude close control of differences in tumour size. Consequently, it is possible that the reduced survival among divorced women may be attributable to a larger tumour at diagnosis in this group of breast cancer patients.

We also found an increased case fatality for cervical cancer among divorced women. At diagnosis, 23.3% of divorced women had stage 1 disease compared with 31.7% of married women ($P=0.03$), and a higher proportion of divorced women had advanced disease ($P=0.02$). After adjustment for these differences, however, a 25% increased death rate persisted among divorced women with cervical cancer, and this corresponds to the findings of Murphy *et al.* (1993).

For lung cancer, it has been suggested that married patients have longer survival owing to earlier stage at diagnosis (Ganz *et al.*, 1991). After adjustment for age and stage at diagnosis, divorced women in our study had a 30% increased risk, and widows had a risk of dying identical to married women.

The prognosis of colorectal cancer largely depends on early detection and skilled surgery (Kronborg, 1993). Kato *et al.* (1992) found that single women, but not divorced or widowed women had shorter survival from colorectal cancer than the married. In our study, stage was uniformly distributed between marital groups, but for widowed patients with colorectal cancer we found an unexpected 2-fold increased risk of dying compared with married women. This association could be a result of chance, however, because of the many statistical comparisons.

Hislop *et al.* (1987) hypothesised that survival may be longer in women who have established strong emotional ties with family and friends. Studies have suggested (Gove, 1973; Jones and Goldblatt, 1986) that having children may be 'protective' for total survival, and this effect may persist into widowhood. We found that cancer patients with children had an overall 20% lower risk of dying than nulliparous women.

With the exception of ovarian cancer, in which the risk of dying was higher among parous than nulliparous women, a lower hazard ratio was found among parous women for all the sites of cancer that we could analyse. We can offer no explanation for the increased death rate among parous women with ovarian cancer, and it may be questionable that the social support hypothesis applies to the finding that parous women have a lower risk of dying than nulliparous women for the other malignancies that could be studied. Parity is an important predictor of the risk and mortality of a number of cancers, and its effect has often been explained in terms of indicating certain hormonal influences. It is less clear that parity may be of similar importance for the prognosis (survival) of cancer, but as an alternative to the social support hypothesis our findings may suggest that parity, as a proxy indicator of endogenous hormones, may be an important predictor of cancer outcomes.

The question has also been raised that the time interval between life change event (divorce or widowhood) and the diagnosis of cancer may be related to survival (Kaprio *et al.*, 1987). We explored this question, but found no evidence of any time-related pattern regarding time since the event and survival from cancer.

Some studies have shown a positive association between socioeconomic status and survival (Farley and Flannery, 1989; Forsén, 1991; Kogevinas *et al.*, 1991), and attributed the beneficial effect of marriage to higher socioeconomic status and better medical treatment. Socioeconomic differences may also reflect differences in stage at diagnosis, host resistance, tumour characteristics and time of diagnosis. However, the egalitarian character of the Norwegian health system makes it unlikely that different treatment opportunities between marital groups play a major role in our results.

One limitation in our study may be the lack of information on cause-specific death. Since the participants are still in middle age, it may be assumed that most deaths would be caused by the cancer, and not be due to other causes. This is in agreement with Tretli *et al.* (1990), who found that the difference between the number of observed and expected deaths among middle-aged Norwegian breast cancer patients was negligible.

Another limitation is the lack of information about cohabitation. Some cohabiting women will therefore be classified as unmarried and, hence, be excluded from the analysis.

Overall, we found that divorced women with cancer had a slightly worse prognosis than married and widowed women. The clearest site-specific result was that widows had an increased death rate from colorectal cancer. Women with children had a generally better prognosis than nulliparous women, and this result may favour the social support hypothesis. Alternatively, it could be attributed to biological effects of parity. For cancers of poor prognosis or advanced stage (Kogevinas *et al.*, 1991; Bjørge *et al.*, 1993), the intrinsic biology is likely to be the most important predictor of death, but for local cancer this study may support the hypothesis that other, not primarily biological, factors (e.g. lifestyle, life changes, social support), may be important (Goodwin *et al.*, 1987) for the outcome of women with cancer. Such factors may include a greater tendency to neglect one's health, patient delay in diagnosis and lower compliance with treatment.

**Acknowledgements**

The technical assistance of Dr Øystein Kravdal (The Central Bureau of Statistics) and Mr Rolf Petter Halle (SINTEF) is greatly appreciated. This study is supported by Grant No. 90098/002 from the Norwegian Cancer Society, and No. 363.91 from the Norwegian Research Council.

**References**

ALTMAN DG. (1991). *Practical Statistics for Medical Research*. Chapman & Hall: London.

BARRACLOUGH J, PINDER P, CRUDDAS M, OSMOND C, TAYLOR I AND PERRY M. (1992). Life events and breast cancer prognosis. *Br. Med. J.*, **304**, 1078–1081.

BJØRGE T, THORESEN SØ AND SKARE GB. (1993). Incidence, survival and mortality in cervical cancer in Norway, 1956–1990. *Eur. J. Cancer*, **29A**, 2291–2297.

BOWLING A. (1987). Mortality after bereavement: a review of the literature on survival periods and factors affecting survival. *Soc. Sci. Med.*, **24**, 117–124.

COOMBS RH. (1991). Marital status and personal well-being: a literature review. *Family Relations*, **40**, 97–102.

EWERTZ M. (1993). Breast cancer in Denmark. Incidence, risk factors, and characteristics of survival. *Acta Oncol.*, **32**, 595–615.

FARLEY TA AND FLANNERY JT. (1989). Late-stage diagnosis of breast cancer in women of lower socioeconomic status: public health implications. *Am. J. Public Health*, **79**, 1508–1512.

FORSÉN A. (1991). Psychosocial stress as a risk for breast cancer. *Psychother. Psychosom.*, **55**, 176–185.

FUNCH DP AND MARSHALL J. (1983). The role of stress, social support and age in survival from breast cancer. *J. Psychosom. Res.*, **27**, 77–83.

GANZ PA, LEE JJ AND SIAU J. (1991). Quality of life assessment. An independent prognostic variable for survival in lung cancer. *Cancer*, **67**, 3131–3135.

GOODWIN JS, HUNT WC, KEY CR AND SAMET JM. (1987). The effect of marital status on stage, treatment, and survival of cancer patients. *JAMA*, **258**, 3125–3130.

GOVE WR. (1973). Sex, marital status, and mortality. *Am. J. Sociol.*, **79**, 45–67.

HELSING KJ AND SZKLO M. (1981). Mortality after bereavement. *Am. J. Epidemiol.*, **114**, 41–52.

HILAKIVI-CLARKE L, ROWLAND J, CLARKE R AND LIPPMAN ME. (1993). Psychosocial factors in the development and progression of breast cancer. *Breast Cancer Res. Treat.*, **29**, 141–160.

HISLOP TG, WAXLER NE, COLDMAN AJ, ELWOOD JM AND KAN L. (1987). The prognostic significance of psychosocial factors in women with breast cancer. *J. Chron. Dis.*, **40**, 729–735.

JACOBS S AND OSTFELD A. (1977). An epidemiological review of the mortality of bereavement. *Psychosom. Med.*, **39**, 344–357.

JONES DR AND GOLDBLATT PO. (1986). Cancer mortality following widow(er)hood: some further results from the Office of Population Censuses and Surveys Longitudinal Study. *Stress Med.*, **2**, 129–140.

JOSEPH JG AND SYME SL. (1982). Social connection and the etiology of cancer: an epidemiological review and discussion. In *Psychosocial Aspects of Cancer*, Cohen J, Cullen JW, Martin LR. (eds.) pp. 151–162. Raven Press: New York.

JOUNG IMA, VAN DE MHEEN H, STRONKS K, VAN POPPEL FWA AND MACKENBACH JP. (1994). Differences in self-reported morbidity by marital status and by living arrangement. *Int. J. Epidemiol.*, **23**, 91–97.

KAPRIO J, KOSKENVUO M AND RITA H. (1987). Mortality after bereavement: a prospective study of 95,647 widowed persons. *Am. J. Public Health*, **77**, 283–287.

KATO I, TOMINAGA S AND IKARI A. (1992). The role of socioeconomic factors in the survival of patients with gastrointestinal cancers. *Jpn J. Clin. Oncol.*, **22**, 270–277.

KOGEVINAS M, MARMOT MG, FOX AJ AND GOLDBLATT PO. (1991). Socioeconomic differences in cancer survival. *J. Epidemiol. Commun Health.*, **45**, 216–219.

KOSKENVUO M, SARNA S AND KAPRIO J. (1979). Cause-specific mortality by marital status and social class in Finland during 1969–1971. *Soc. Sci. Med.*, **13A**, 691–697.

KRAUS AS AND LILIENFELD AM. (1959). Some epidemiologic aspects of the high mortality rate in the young widowed group. *J. Chron. Dis.*, **10**, 207–217.

KRAVDAL Ø, GLATTRE E AND HALDORSEN T. (1991). Positive correlation between parity and incidence of thyroid cancer: new evidence based on complete Norwegian birth cohorts. *Int. J. Cancer*, **49**, 1–6.

KRONBORG O. (1993). Staging and surgery for colorectal cancer. *Eur. J. Cancer*, **29A**, 575-583.

LUND E. (1981). Pilot study for the evaluation of completeness of reporting to the Cancer Registry. In *Incidence of Cancer in Norway 1978*, p 11. Cancer Registry of Norway: Oslo.

MARX JL. (1985). The immune system 'belongs in the body'. *Science*, **227**, 1190-1192.

MASTEKAASA A. (1993). Does marriage protect the individual: the case of unemployment and subjective well-being. *J. Sociol. (Sosiologisk Tidsskrift)*, **3**, 193-211.

MELLSTRÖM D, NILSSON Å, ODÉN A, RUNDGREN Å AND SVANBORG A. (1982). Mortality among the widowed in Sweden. Scand. *J. Soc. Med.*, **10**, 33-41.

MURPHY MFG, GOLDBLATT PO AND MANT D. (1993). Marital stability and cancer of the uterine cervix: changing patterns in post-war Britain. *Int. J. Epidemiol.*, **22**, 385-392.

NAYERI K, PITARO G AND FELDMAN JG. (1992). Marital status and stage at diagnosis in cancer. *NY State J. Med.*, **92**, 8-11.

NEALE AV, TILLEY BC AND VERNON SW. (1986). Marital status, delay in seeking treatment and survival from breast cancer. *Soc. Sci. Med.*, **23**, 305-312.

PARKES CM, BENJAMIN B AND FITZGERALD RG. (1969). Broken heart: a statistical study of increased mortality among widowers. *Br. Med. J.*, **1**, 740-743.

PETO R, PIKE MC, ARMITAGE P, BRESLOW NE, COX DR, HOWARD V, MANTEL N, McPHERSON K, PETO J AND SMITH PG. (1977). Design and analysis of randomized clinical trials requiring prolonged observation of each patients. II. Analysis and examples. *Br. J. Cancer*, **35**, 1-39.

RAMIREZ AJ, CRAIG TKJ, WATSON JP, FENTIMAN IS, NORTH WRS AND RUBENS RD. (1989). Stress and relapse of breast cancer. *Br. Med. J.*, **298**, 291-293.

REES WD AND LUTKINS SG. (1967). Mortality of bereavement. *Br. Med. J.*, **4**, 13-16.

SEEMAN TE, KAPLAN GA, KNUDSEN L, COHEN R AND GURALNIK J. (1987). Social network ties and mortality among the elderly in the Alameda County study. *Am. J. Epidemiol.*, **126**, 714-723.

SUSSER M. (1981). Widowhood: a situational life stress or a stressful life event? *Am. J. Public Health*, **71**, 793-795.

TRETLI S, HALDORSEN T AND OTTESTAD L. (1990). The effect of pre-morbid height and weight in the survival of breast cancer patients. *Br. J. Cancer*, **62**, 299-303.

WARD AWM. (1976). Mortality of bereavement. *Br. Med. J.*, **i**, 700-702.

YOUNG M, BENJAMIN B AND WALLIS C. (1963). The mortality of widowers. *Lancet*, **ii**, 454-456.